# Genetic testing for hereditary cancer syndromes: patient recommendations for improved risk communication

**DOI:** 10.1111/hex.13062

**Published:** 2020-04-27

**Authors:** Samantha Pollard, Steve Kalloger, Deirdre Weymann, Sophie Sun, Jennifer Nuk, Kasmintan A. Schrader, Dean A. Regier

**Affiliations:** ^1^ Canadian Centre for Applied Research in Cancer Control, Cancer Control Research BC Cancer Vancouver BC Canada; ^2^ School of Population and Public Health University of British Columbia Vancouver BC Canada; ^3^ Department of Pathology & Laboratory Medicine University of British Columbia Vancouver BC Canada; ^4^ Hereditary Cancer Program BC Cancer Vancouver BC Canada; ^5^ Division of Medical Oncology BC Cancer Vancouver BC Canada; ^6^ Department of Medicine Faculty of Medicine University of British Columbia Vancouver BC Canada; ^7^ Department of Medical Genetics Faculty of Medicine University of British Columbia Vancouver BC Canada; ^8^ Department of Molecular Oncology BC Cancer Vancouver BC Canada

**Keywords:** family communication, genetic counselling, genetic testing, hereditary cancer syndromes, medical decision making, risk communication

## Abstract

**Background:**

Multi‐gene panel testing is replacing single‐gene testing for patients with suspected hereditary cancer syndromes. The detection of a hereditary cancer syndrome allows tested individuals to initiate enhanced primary and secondary prevention efforts—where available—with a view to reduce disease burden. Current policy prevents testing programmes from communicating genetic test results with potentially affected family members, yet it is well documented that tested individuals face multiple challenges in initiating such discussions with relatives.

**Objective:**

In response to this challenge, we sought patient recommendations about how to improve genetic risk communication to enhance interfamilial discussions about primary and secondary disease prevention.

**Design:**

We conducted 25 semi‐structured interviews with individuals who received genetic testing through British Columbia’s Hereditary Cancer Program between 2017 and 2018. Interviews were professionally transcribed and analysed using a constant comparative approach.

**Results:**

Participants described difficulty engaging in conversations with relatives who were resistant to receiving genetic risk information, when communicating with younger relatives and where participants reported strained familial relationships. Participants recommended that testing facilities provide a summary of results and implications and that resources be made available to prepare patients for challenging discussions with family members.

**Discussion:**

Our study demonstrates that individuals undergoing genetic testing for suspected hereditary cancer syndromes would benefit from additional supportive resources alongside genetic counselling. Providing this on‐going support will enhance the accurate and transparent communication of risk to facilitate the uptake of cascade testing and enhanced prevention strategies.

## 1 INTRODUCTION

Genetic testing using next‐generation sequencing technologies for the diagnosis of hereditary cancer syndromes is increasingly transitioning from research to clinical settings. The clinical application of multi‐gene panels is intended to guide decisions about enhanced primary and secondary prevention strategies as well as genetics‐informed treatment options. Due to the hereditary nature of certain cancer syndromes such as Lynch, Li‐Fraumeni and hereditary breast and ovarian cancer syndromes, test results carry implications for the first tested individual in the family—probands—as well as their genetic family members. Communicating accurate genetic risk information allows potentially affected family members to undergo testing and—where appropriate—benefit from enhanced prevention strategies.^1^, ^2^, ^3^, ^4^, ^5^

Current legislation within Canada and the United States does not require probands to discuss genetic test results with family members.^6^ Due to privacy concerns, health‐care providers are unable to contact probands’ relatives directly.^1^ While the communication of genetic test results to genetic relatives is not legally required, accurately relaying information about familial risk carry both personal and health systems implications. A wealth of evidence suggests that communicating genetic risk information is a complex process affected by both informational and interpersonal barriers and subjects to substantial individual variation.^2^, ^7^, ^8^, ^9^, ^10^, ^11^, ^12^, ^13^, ^14^, ^15^ Patient‐reported barriers to communication about genetic risk include informational complexity, motivation, family culture, as well as predicted and experienced familial reactions.^7^, ^8^, ^11^, ^16^, ^17^, ^18^

In response to reported barriers, health‐care providers offer strategies such as allocating genetic counselling time to addressing family dynamics, as well as the provision of family letters and testing facility contact information to relatives.^18^, ^19^ In addition, testing institutions provide guidance to encourage communication with potentially affected family members.^20^, ^21^ Despite such efforts, rates of cascade testing remain minimal, limiting the population benefit attributable to genetic testing for familial cancers.^12^, ^22^

Tested individuals are the vehicle by which information about hereditary cancer susceptibility is relayed to family members. For this reason, there exists an unmet need to determine how to enhance the communication process, from the perspectives of those who have undergone testing. To date, there is a lack of patient‐directed guidance to mitigate challenges to communicating genetic risk to relatives. High‐quality patient decision support techniques present an opportunity to enhance shared decision making and supplement genetic counselling sessions, but do not yet exist for the purposes of enhancing interfamilial communication about hereditary cancers.^23^, ^24^ Decision support techniques have been shown to increase patient‐physician communication; promote accurate knowledge; clarify values; resolve decisional conflict; and increase decisional satisfaction.^25^, ^26^, ^27^, ^28^, ^29^, ^30^, ^31^ In context to genetic testing for hereditary cancers, it is critical to not only understand, but *address* barriers to effective communication. Enhancing communication between patents and relatives will increase the uptake of cascade testing. Determining what patients need to improve the communication process will help to meet this overarching objective will help to ensure equitable access to prevention strategies for high‐risk families.

### 1.1 Objectives

The current study was initiated in response to an unmet need to identify patient‐guided strategies to improve communication about hereditary cancer susceptibility. This work represents the preliminary phase of a larger investigation to develop a patient‐values informed decision support technique for hereditary cancer susceptibility genetic testing. The qualitative work was conducted specifically to inform the content of the decision support tool in the form of an e‐health application developed for feasible implementation. Here, we report on the aspect of patient interviews that pertain to communicating genetic testing information to family members.

This study was conducted in collaboration with the British Columbia (BC) Cancer’s Hereditary Cancer Program (HCP), the sole provider of publicly funded hereditary cancer genetic counselling and testing services across BC and the Yukon.^32^ The protocol and all study documentation were approved by the University of British Columbia BC Cancer Behavioural Research Ethics Board (ethical approval # H18‐00644). Authors have followed COREQ guidelines to report this investigation.^33^

## 2 MATERIALS AND METHODS

### 2.1 Patient partner engagement

Our patient‐oriented approach involved on‐going consultation with a patient partner diagnosed with a hereditary cancer syndrome. Our patient partner was actively involved in protocol development, review of all study documentation, interview study design, interpretation of findings and review of research outputs. She attended all research team meetings either via teleconference or in‐person.

### 2.2 Interview guide development and piloting

The study team developed an interview guide to explore patient experiences with the process of genetic testing and communicating test results with family members. The content of the interview guide was informed by previous qualitative studies examining patient opinions about genetic testing, the broad semi‐structured interview guide development literature, as well as consultation with our patient partner.^34^, ^35^, ^36^ We applied a flexible framework to address the general topics considered important for discussion. We framed discussions around the potential for a decision support tool by asking participants what could have helped to facilitate more effective communication with relatives. Following initial development, the interview guide was piloted extensively within the research team, including our patient partner. The purpose of piloting was to ensure adequate coverage of content; to estimate interview length; and to identify and address potential researcher bias (Table 1). Through consultation with the research team and our patient partner, we aimed to ensure that preconceived expectations regarding barriers to communication with family were not being relayed through phrasing of interview prompts. The interview guide was finalized, following research team consensus. Interview guide development methods are consistent with published recommendations.^34^

**Table 1:** Interview topic guide

Topic	Selected semi‐structured interview question	Prompt (optional)
Decision‐making process	What kind of information do you think is important to have before deciding whether or not to have a genetic test?	Is there anything that you know now that you wish you had known before you made the decision? Which of these issues is the MOST important to you?
	How do you think that information should be presented?	In person by a genetic counselor? Online?
Experience with the return of results	Can you tell me about what it was like to receive the results of your genetic test?	Is there anything that could have been done to make the results easier to understand?
Experience communicating with family	How prepared did you feel, to receive the results of your genetic testing?	Did you have enough information? Would you have preferred to have additional information prior to receiving those results?
	Did you choose to share the results of your genetic test? Why or why not?	With which family members did you share the results? Is there anything that could have made it easier to share that information with (family member)?

### 2.3 Participant eligibility and recruitment

Patients eligible for this study were 19 years or older, received carrier or index genetic test results between 2017 and 2018, had previously consented to be contacted for future research studies and were able to complete an interview in English. Index testing occurs when the proband is tested in the absence of a previously identified hereditary cancer syndrome in his or her family. Carrier testing is undertaken when a familial cancer syndrome has already been identified in the family, and typically, an asymptomatic individual is tested for the presence of known syndrome‐specific pathogenic (disease causing) variants.

To maximize participation, two recruitment approaches were taken. First, we identified a list of individuals having recently undergone genetic testing and had provided written consent to be contacted for future research. Potentially eligible participants were identified through the Hereditary Cancer Program database. Following the identification of eligible individuals, the study coordinator (SP) distributed a study invitation and consent letter via post. Non‐responders were contacted via telephone to assess interest after two weeks. Second, one investigator (SS) approached eligible patients in clinic to ascertain interest in the study. The study coordinator followed up with individuals who expressed interest in participating. Investigators applied a maximum variation sampling technique to ensure diversity in age, sex and personal experience with cancer. Participant recruitment continued until two reviewers (SP and SK) agreed that data saturation had been achieved.

### 2.4 Study process

A female PhD health researcher (SP) with qualitative research experience conducted all interviews. Participants were provided the option to be interviewed over the telephone or in‐person at the BC Cancer Research Centre in Vancouver, BC. The interviewer had no previous interaction with participants other than communicating the purpose of the research and obtaining consent in advance of the interviews. To the best of our knowledge, participants had no prior familiarity with the interviewer. No interviewer characteristics were provided beyond information about her participation in the research project. All interviews were audio‐recorded following written participant consent. The interviewer took minimal notes during the interview and documented field notes immediately following. For in‐person interviews, only the interviewer and participant were present in the room. A distress protocol was maintained during each interview.^37^ Participants completed a brief demographics questionnaire prior to each interview. Each participant was mailed an honorarium of CAD$75.00 following the interview.

All interview transcripts were professionally transcribed, de‐identified and reviewed for accuracy prior to initiating the qualitative analysis. Transcripts and field notes were maintained on the research group’s password‐protected secured drive with access limited to researchers listed on the ethics approval. Interview transcripts were not returned to participants.

Two coders (SP and SK) applied a grounded theory approach to the qualitative analysis, using constant comparison for the development of the code book.^38^ Analytic codes were identified inductively to ensure that major themes emerged from the data rather than through a priori expectations. Interviews were coded in batches of two using QSR International’s NVivo 12 qualitative data analysis software.^39^ Coders discussed in vivo codes after each reviewing two transcribed interviews, identifying areas of disagreement and revising the code book accordingly. This process continued until both coders were satisfied with their agreement and the code book. SP revised and maintained the code book throughout the iterative process of interviewing and analysis. To ensure consistency, reviewers coded 30% of the interview transcripts independently and in duplicate. SP coded the remaining interviews. Themes that arose through the analytic process were not verified by interview participants.

## 3 RESULTS

Between June and September 2018, 49 patients were invited to participate in the interview study. Of these, 26 consented to participate and 25 interviews were completed (51% overall response). A single interviewer (SP) conducted 21 telephone and 4 in‐person interviews. Interviews lasted approximately 45 minutes with a range of 30‐70 minutes.

The majority of participants were female (64%), married (72%), educated beyond high school equivalence (72%) and had a personal history of cancer (76%). Participants’ age ranged from 32 to 78 years. Among patients with a self‐reported personal history of cancer, 70% (n=14) had experienced either breast or colon cancer, consistent with the distribution of patients who are referred to the HCP. Other diagnoses included lung cancer, malignant melanoma, testicular, kidney, bowel, uterine and endometrial cancer. Participants with a personal history of cancer reported between 1 and 3 primary cancer types.

According to participant report, 21 underwent index testing and four participants were carrier tested. Just under half (43%) of participants reported having received a pathogenic variant. Three of four patients (75%) who underwent carrier testing reported that they received an uninformative result. A total of 10 participants (40%) reported the return of a variant of unknown significance or an inconclusive finding (see Table 2).

**Table 2:** Participant demographics (N=25)

		
Participant characteristic	**N**	**%**
Female	16	64
Mean age (range)	53 (32‐78)	
Marital status		
Married/ civil partnership	18	72
Single	3	12
Divorced/ separated	3	12
Widowed	1	4
Education		
≤ High school	7	28
Non‐university certificate	9	36
University degree	9	36
Employment		
Paid	12	48
Retired	7	28
Long‐term disability	6	24
Cancer Site^a^		
Breast	9	36
Colon	5	20
Melanoma	2	8
Testicular	1	4
Renal	2	8
Lung	2	8
Endometrial	1	4
Ovarian	1	4
Participant reported test result		
Index negative or carrier uninformative	15	60
Index or carrier pathogenic	10	40
Variant of unknown significance/inconclusive finding	10	60

a Categories are not mutually exclusive.

## 4 Communication patterns

Across 25 participants, there was substantial heterogeneity regarding experiences communicating genetic test results to family. While the majority had made at least one attempt at communication, a small minority (n=2) had chosen not to disclose the fact that they had undergone testing. One participant had made one attempt with the intention of broaching the topic again. Only one participant with a reported‐pathogenic variant explicitly stated that she had not attempted communicating her results with genetic family members. We also identified variation in terms of the timing of communication, often informed by participants’ comfort level with raising the topic of genetic testing and disease status. Although some participants reported speaking with family members throughout the testing process and before the return of results, others waited until they had received their results to attempt discussions.

Among patients who reported having initiated conversations with family either before or after testing, 7 referenced conversations with first‐degree relatives (eg children, siblings and parents) and an additional 4 participants specifically referenced conversations with second‐degree relatives (eg cousins, nieces and nephews). Eight participants specifically stated that at least one reason for receiving testing was to inform family members about their cancer risk. Other reasons for testing included a desire to inform treatment and prevention options, to assist in making family planning decisions, as well as general life planning.

## 5 Summary of major themes

Interview participants described a variety of experiences communicating genetic risk to family members. Communication challenges were highly contextualized given initial attempts at discussions about test results, relatives’ perceptions about cancer risk, the presence of a family history of cancer, family culture, and interpersonal relationships with relatives. Here, we report barriers and facilitators identified through the qualitative synthesis, followed by a discussion of participant recommendations for facilitating constructive risk communication (Table 3).

**Table 3:** Barriers to communicating genetic risk with family members

Major analytic theme	Quote #	Supporting quote
Family culture	1	“Like, the first thing I was kind of annoyed at my extended family for not telling us anything. But we lived in a kind of family who has a culture that you don't tell stuff that's personal. And so, man oh man, it would have been really good to know about that, but that's nothing to do with institutions. That's a family culture problem, the hush‐hush.” (004)
	2	“I don’t communicate with my family”. (024)
	3	“…it's hard because my mom is not living with me, she is back home…So, it's hard to tell them what's really going on. Our culture is different from here…They hear you have cancer…I know I'm going to die, but it's hard for them and it's hard for me”. (001)
	4	“We're very open to talking to one another about everything in our lives, and so when we share something like heredity and DNA, then it just sort of confirms that we are definitely part of a family”. (003)
Communicating with young relatives	5	“I have two sons, grown‐up sons with children of their own. And I told them. It was hard to tell them…I remember myself at their age. Things like that meant nothing to me…” (014)
	6	“I don't know how seriously they take it. You know, even with the melanoma…I'm always going on about sunscreen, and being careful and wearing hats, and… I don't think they pay that much attention or take it that seriously… I find that, you know, that's the thing about young people thinking they're infallible perhaps’. (018)
	7	“The conversation with my brother was… awkward a little bit…I don’t really think he fully understands what it might mean for him down the road if he decides to have children”. (009)
Resistant family members	8	“So I just tried to explain to her that it's good to know, and now you will be able to keep on top of it with screening and so on. So it's good information to have. And the other daughter, of course, feels the opposite. She feels not knowing would be better than having the stress of knowing”. (012)
	9	“She's one of these people that isn't wanting to know too many medical details”. (021)
	10	“I think it scared them when I got cancer because they saw what happened to my mom. So when it came down to the genetics, they didn’t want to think about it. Especially my oldest and she’ll probably never, ever”. (023)
	11	And my brother reacts strongly to all this. When I had the…cancer and I told him, he said, “Oh, they always make mistake. You have nothing”. (014)
	12	“I worry more about my nieces and my nephews. And I don't know what to do. If I should tell them or not…It's like putting a bowling ball in their lives. I don't know how to do it”. (014)
	13	“So my brother's wife was a little resistant. She was like, "If you have it I'm not telling my daughters…I don't want to start freaking them out for the future." But to me that was a poor decision, but I guess that leads to some other moral issues. Do you go behind their backs and tell their daughter…?’ (006)
Familiarity with discussing cancer	14	“She's always known, all along, you know, that this may be passed on, and that we will end up having this discussion”. (017)
	15	“I was in touch with the other [siblings] as I was having the testing done, and I said, "This could ‐‐ you know, you could possibly ‐‐ if this test comes back positive, there is an option that you guys could be tested and see if it stops here." And they were all onboard with that. None of them didn't want to know. They all wanted to know so that they could take possible actions to stop it. And two of my siblings have female offspring, and they're worried about them having the breast cancer factors. And so they're going through the testing right now. And I was informed that if it's negative for them, then it doesn't go past that generation”. (002)
	16	“That's maybe one of the sad parts, when there's enough people in the family that have had cancer that ‐‐ I guess they sort of accept it as a reality that, you know, the likelihood of people getting cancer is quite high. So they're sort of familiar with the process”. (015)
	17	It was a little challenging initially just telling my parents, because both of them didn’t want it to be from them. So, there was a bit of tension. And a little bit of guilt from both of them, and that was really tough. 009

### 5.1 Family culture and interpersonal relationships

Interpersonal family relationships and culture played an important role in participant discussions about initiating conversations, and their perceptions about the success of attempts at communication. Participants described strained or distant relationships as well as family cultures wherein sensitive subjects were not discussed (quote 1‐3). Although some participants expressed frustration about the lack of risk communication with relatives (quote 1), a minority appeared unbothered and unmotivated to overcome interpersonal barriers to communication (quote 2). In one case, family culture and perceptions about cancer were explicitly discussed as a primary barrier to initiating conversations about genetic test results.

### 5.2 Fear, anxiety and disinterest

Participants described relatives who would not engage in discussions about genetic risk as well as those who explicitly stated that they did not want to be informed about test results. Reasons for not wanting to receive risk information were multifold. For example, some characterized family members as fearful, overwhelmed or disinterested in health or genetic information (quotes 5 and 6). Others characterized family members as lacking interest in medical information (quote 9), as being distrustful of the health‐care system and health‐care providers (quote 11), or holding the preconceived opinion that cancer cannot be prevented (quote 13). Throughout these conversations, participants did not describe a lack of understanding of their own test results such that they were challenged by how to communicate risk and the value of cascade testing effectively. Rather, participants described feeling ill‐equipped to overcome relatives’ unwillingness to engage in meaningful and constructive discussions.

In a minority of instances, participants described specific challenges related to communicating test results to young family members. These conversations were met with the perception that young adult relatives considered themselves to be at an inherently low risk despite the presence of variant hereditary cancer syndrome in the family. One participant (quote 5) found it difficult to communicate information about her pathogenic *BRCA* variant to her brother and children, each of whom had children of their own. This sentiment was shared by other participants when relaying information with younger family members (quote 5‐7). In each of these discussions, participants expressed marked distress about the difficulty of ensuring that young family members would understand the implications of test results as well as the importance of taking preventative actions to reduce their own cancer risk.

Participants who experienced strained conversations with close family members struggled with the decision to inform second‐degree relatives. In two specific conversations (quotes 12 and 13), participants expressed a lack of clarity about the duty to inform family members. Although the duty to inform was discussed in a small subset of interviews, the topic was met with substantial distress and confusion regarding who should be informed, and how discussions with resistant family members should be broached. Participants whose attempts to relay genetic risk information were stonewalled by resistant family members expressed considerable frustration.

### 5.3 Family history of cancer

The presence of a known family history of cancer served to both facilitate and stifle constructive conversations. As shown in quotes 8, 9, 10 and 13, cancer was at times perceived as something fearful about which family members were unwilling to engage. One participant described her perception that that the detection of a hereditary cancer syndrome would be too much for her daughters to handle given her family’s recent history of multiple cancer diagnoses (quote 10)*.* Other times, the presence of a known family history of cancer helped to facilitate open and constructive discussions (quotes 14 and 15). In such instances, the presence of a strong family history of cancer was described as a common topic of conversation within families or as a feature that brought family members closer together (quotes 4).

## 6 Participant recommendations

Following discussions about communicating genetic risk to family members, participants discussed ways in which an adjunct to genetic counselling could improve this process. Recommendations were not framed specifically in relation to the potential for an e‐health app. Rather, participants discussed recommendations in terms of broad strategies that would support the process of communication (quotes 18‐23). Recommendations included a lay summary of test results and familial implications, alongside practical advice to facilitate constructive conversations. Participants recommended the potential for a lay summary of genetic test results and implications directed to family members. In some cases, participants reported that this could be used to supplement conversations with relatives (quotes 19 and 20). Participant perceived that documentation provided by testing programmes would facilitate and validate conversations and increase the likelihood that family members would initiate prevention strategies. Other participants recognized that a written report could be used in situations where family members reside in geographically disparate locations or within families where communication about sensitive subjects was particularly challenging (quote 19).

The desire for practical advice about communication with family was articulated using multiple examples, such as the ability to connect with previously tested individuals to illustrate what successful conversations with family members might look like (quote 21). Participants spoke about the desire for examples or advice about how to broach the topic of genetic risk (quote 22 and 23). Among individuals who faced resistant family members, there was a lack of confidence about how to accurately relay the information in a manner that would be sensitive to the potential for worry, upset or guilt (quote 18). A key feature of participant recommendations was to better prepare probands for resistant family members and to provide them with the ability to manage negative reactions. Participants sought resources to prepare them for potentially difficult discussions, as well as on‐going support following unsuccessful attempts at communication (Table 4).

**Table 4:** Participant recommendations

Recommendation	Quote #	Supportive quote
Lay summary provided by testing facility	18	“…And explain that the rates are low even among those under 50 or 60. But still to go through that information, and then have it so that they can sort of read it. So something that has like a basic definition of Lynch syndrome, a description of sort of the incidence rate of it, what it would mean in terms of passing that on to your children, and what it would mean for siblings, other family members. Because I know that while I was going through the testing my sister was, you know, stressed out wondering if she should be getting checked out too. And it turned out all to be negative in my case, but it would just be good to have that information up front for people…” (005)
	19	“Something to send to the family members…what the results were, what this means for them, and, you know, what the next step is for them…” (017)
	20	“For family members who you may not even be that close to get the message, sometimes having a form is more helpful, having to call everyone or email…” (018)
Advice for communicating with family	21	“Other people's stories. That maybe, to know what they experience. I think I looked at something like that on the internet, but I would like to have something that is brought by the system, by the medical system where people say this is what happened when I told my children or, you know, my siblings and their reaction. And I think something like that would have helped. To be prepared of what the possibilities are” (014)
	22	“…you have to have the conversation with them. You can't just send them an e‐mail…So, something maybe around coaching people on how to tell family might be a little bit more useful”. (009)
	23	“I think it would have been good to sort of address that piece, like how to approach your children about ‐‐ regardless if they’re positive or negative…a pamphlet on how to talk to children?”(020)

## 7 DISCUSSION

Although previous investigations have elicited genetic counsellor practice patterns for enhancing family communication, to our knowledge, no patient‐reported recommendations to directly inform the development of a decision support tool exist.^19^ Our study provides a novel contribution to the literature by providing patient‐reported recommendations to mitigate experienced barriers to effective communication. Our results further establish the argument that interpersonal barriers such as family dynamics substantially impact communication patterns.^40^

Throughout discussions, participants acknowledged the potential for worry and anxiety among family members. Participants discussed waiting to speak with family members until results had been returned in an effort to mitigate unnecessary concern. Participants also discussed the impact that worry and anxiety had on familial reactions to positive test results. Consistent with recent literature, these findings support the development of methods and resources to ensure that genetic risk information is relayed in a manner that will not overwhelm or overburden potentially affected relatives.^4^

Our results further substantiate the individualized nature of successful communication. While the presence of a family history of cancer was at times a strong motivator and facilitator for effective communication, other participants experienced opposing reactions from relatives. This finding speaks to the need for an individualized approach to preparing patients for discussions, given their personal and familial experiences, perceptions and expectations. The implementation of an *individualized* approach to encouraging discussions about genetic risk best aligns with patient preferences and experiences. Given the communication challenges faced by our participants, these findings further suggest that probands may be accepting of direct contact of potentially affected relatives by testing facilities, where conversations are challenging or infeasible.^5^ Further work is warranted to address proband and relatives’ acceptance of a direct contact approach.

As evidenced here, genetic counselling for hereditary cancer testing does not always adequately prepare patients to relay their results to family members. In resource‐scarce health systems, genetic counselling sessions are typically limited in length and frequency, owing partially to a shortage of available counsellors. A substantial gap exists between the need for and availability of genetic counsellors to adequately guide patients through complex decision making.^41^, ^42^ For these reasons, it is infeasible that genetic counsellors bear the burden of providing additional individualized and on‐going support for each of their patients in an effort to resolve this on‐going challenge. Our participants favour the development of supportive resources to provide guidance for genetic risk communication, as an adjunct to genetic counselling.

### 7.1 Limitations

The results of this work should be interpreted alongside limitations. Firstly, responses to the interview were self‐reported and therefore subject to reporting bias. To mitigate this potential bias, participants were informed that only de‐identified information would be reported and that all interview responses would remain confidential. Consent documentation provided assurance that responses would not impact clinical care.

Further, we did not validate participant reported test results with individual patient test result reports. For this reason, we are unable to determine whether participants accurately understood their test results. While we report briefly on self‐reported results to frame discussions, the focus of this work centred on experiences communicating test results with relatives. Recognizing this limitation, in the second stage of this study—following the development of the decision support tool—we plan to validate self‐reported results with testing reports to determine the presence of recall bias or recall inaccuracies.

Finally, our sample consisted of participants who had previously consented to be contacted for future research. The subset of individuals interested in research participation may differ systematically from the total population who underwent genetic testing. Despite the potential for selection bias, we sought to ensure a diversity of perspectives and experiences were captured, and recruitment continued until thematic saturation was reached. Qualitative research studies such as this do not seek sample representativeness and generalizability of findings. Given the diversity of communication experiences captured in this interview study, we are able to report on a substantial heterogeneity of experiences and challenges communicating genetic information to family.

## 8 CONCLUSIONS

Successful communication between probands and their family members is a highly individualized experience informed by multiple interpersonal factors. Our findings support the development of improved resources to assist patients through the entire trajectory of genetic testing. This on‐going support will ensure patients have the motivation, self‐efficacy, and informational resources for successful communication with their families. We further identify a need for practical guidance about how to broach conversations, and how to manage family dynamics when discussions are unsuccessful. While genetic counsellors have a responsibility to provide patients with adequate information that prepares them for constructive risk communication, they are facing time constraints in publicly funded health‐care systems. Additional supports that reduce genetic counsellor burden and enhance the familial communication process will better enable cascade testing programmes to benefit at‐risk families.

## CONFLICT OF INTEREST

The authors declare that they have no conflict of interest.

## Data Availability

Individual patient‐level data that support this research carry the potential to identify participants. To align with our ethics approvals and maintain patient privacy and confidentiality, research data are not shared.
